# Supplementary material for: Activity of Patchouli and Tea Tree Essential Oils against Staphylococci Isolated from Pyoderma in Dogs and Their Synergistic Potential with Gentamicin and Enrofloxacin
Source: Animals (Basel). 2023 Apr 7;13(8):1279. doi: 10.3390/ani13081279 (PMC10134980; doi:10.3390/ani13081279)
Supplement: Supplementary file 1 [file animals-13-01279-s001.zip › animals-2274255-supplementary.pdf]

Table S1. Checkerboard analysis with final interactions when the essential oil concentration is expressed in mg/mL instead % v/v (best match within triplicate; differences to Table 3. are highlighted in **bold** and red).

antimicrobial agent	patchouli oil				tea tree oil			
	MIC_i	MIC_c	FIC	ΣFIC [interaction]	MIC_i	MIC_c	FIC	ΣFIC [interaction]
***Staphylococcus aureus* ATCC 25923**								
oil (mg/mL)<br>gentamicin (µg/mL)	**2.4**<br>1	**0.08**<br>0.25	0.03**3**<br>0.25	0.28**3**<br>synergy	**22.4**<br>1	**0.7**<br>0.25	0.03**1**<br>0.25	0.28**1**<br>synergy
oil (mg/mL)<br>enrofloxacin (µg/mL)	**1.2**<br>0.125	**1.2**<br>0.125	1<br>1	2.0<br>none	**11.2**<br>0.125	**11.2**<br>0.125	1<br>1	2.0<br>none
***Staphylococcus aureus* Sa 1**								
oil (mg/mL)<br>gentamicin (µg/mL)	**4.8**<br>2	**1.2**<br>1	0.25<br>0.5	0.75<br>additive	**44.8**<br>0.5	**11.2**<br>0.125	0.25<br>0.25	0.5<br>synergy
oil (mg/mL)<br>enrofloxacin (µg/mL)	**4.8**<br>8	**2.4**<br>4	0.5<br>0.5	1.0<br>additive	**44.8**<br>8	**11.2**<br>1	0.25<br>0.125	0.375<br>synergy
***Staphylococcus aureus* Sa 2**								
oil (mg/mL)<br>gentamicin (µg/mL)	**4.8**<br>1	**1.2**<br>0.5	0.25<br>0.5	0.75<br>additive	**89.6**<br>0.5	**11.2**<br>0.0625	0.125<br>0.125	0.25<br>synergy
oil (mg/mL)<br>enrofloxacin (µg/mL)	**2.4**<br>4	**2.4**<br>4	1<br>1	2.0<br>none	**44.8**<br>4	**44.8**<br>4	1<br>1	2.0<br>none
***Staphylococcus aureus* Sa 3**								
oil (mg/mL)<br>gentamicin (µg/mL)	**4.8**<br>16	**1.2**<br>4	0.25<br>0.25	0.5<br>synergy	**22.4**<br>8	**5.6**<br>2	0.25<br>0.25	0.5<br>synergy
oil (mg/mL)<br>enrofloxacin (µg/mL)	**2.4**<br>32	**2.4**<br>32	1<br>1	2.0<br>none	**44.8**<br>32	**44.8**<br>32	1<br>1	2.0<br>none
***Staphylococcus aureus* Sa 4**								
oil (mg/mL)<br>gentamicin (µg/mL)	**2.4**<br>0.5	**1.2**<br>0.0625	0.5<br>0.125	0.625<br>additive	**22.4**<br>0.5	**2.8**<br>0.0625	0.12**5**<br>0.125	0.25**0**<br>synergy
oil (mg/mL)<br>enrofloxacin (µg/mL)	**2.4**<br>0.25	**2.4**<br>0.25	1<br>1	2.0<br>none	**11.2**<br>0.125	**11.2**<br>0.125	1<br>1	2.0<br>none
***Staphylococcus aureus* Sa 5**								
oil (mg/mL)<br>gentamicin (µg/mL)	**2.4**<br>0.25	**0.6**<br>0.125	0.25<br>0.5	0.75<br>additive	**22.4**<br>0.25	**5.6**<br>0.032	0.25<br>0.128	0.378<br>synergy
oil (mg/mL)<br>enrofloxacin (µg/mL)	**2.4**<br>1	**2.4**<br>1	1<br>1	2.0<br>none	**11.2**<br>0.5	**11.2**<br>0.5	1<br>1	2.0<br>none
***Staphylococcus aureus* Sa 6**								
oil (mg/mL)<br>gentamicin (µg/mL)	**1.2**<br>0.125	**0.15**<br>0.032	0.12**5**<br>0.256	0.38**1**<br>synergy	**11.2**<br>0.125	**1.4**<br>0.016	0.12**5**<br>0.128	0.25**3**<br>synergy
oil (mg/mL)<br>enrofloxacin (µg/mL)	**1.2**<br>0.125	**0.3**<br>0.0625	0.25**0**<br>0.5	0.75**0**<br>additive	**11.2**<br>0.125	**5.6**<br>0.0625	0.5<br>0.5	1.0<br>additive
***Staphylococcus aureus* Sa 7**								
oil (mg/mL)<br>gentamicin (µg/mL)	**2.4**<br>0.125	**0.6**<br>0.032	0.25<br>0.256	0.506<br>additive	**11.2**<br>0.0625	**2.8**<br>0.032	0.25**0**<br>0.512	0.76**2**<br>additive
oil (mg/mL)<br>enrofloxacin (µg/mL)	**1.2**<br>0.0625	**0.6**<br>0.032	0.5<br>0.512	1.012<br>additive	**5.6**<br>0.125	**5.6**<br>0.125	1<br>1	2.0<br>none
***Staphylococcus pseudintermedius* ED99**								
oil (mg/mL)	**2.4**	**0.3**	0.12**5**		**22.4**	**2.8**	0.12**5**	

antimicrobial agent	patchouli oil				tea tree oil			
	MIC_i	MIC_c	FIC	ΣFIC [interaction]	MIC_i	MIC_c	FIC	ΣFIC [interaction]
gentamicin (µg/mL)	0.5	0.032	0.064	0.189	0.5	0.064	0.128	0.253
oil (mg/mL)	1.2	0.08	0.067	0.567	11.2	11.2	1	2.0
enrofloxacin (µg/mL)	0.25	0.125	0.5	additive	0.125	0.125	1	none
***Staphylococcus pseudintermedius* Sps 1**								
oil (mg/mL)	4.8	0.6	0.125	0.375	44.8	5.6	0.125	0.375
gentamicin (µg/mL)	64	16	0.25	synergy	32	8	0.25	synergy
oil (mg/mL)	2.4	0.15	0.0625	0.563	11.2	0.7	0.0625	0.313
enrofloxacin (µg/mL)	32	16	0.5	additive	64	16	0.25	synergy
***Staphylococcus pseudintermedius* Sps 2**								
oil (mg/mL)	4.8	1.2	0.25	0.75	11.2	1.4	0.125	0.375
gentamicin (µg/mL)	64	32	0.5	additive	32	8	0.25	synergy
oil (mg/mL)	2.4	0.15	0.0625	0.563	11.2	11.2	1	2.0
enrofloxacin (µg/mL)	64	32	0.5	additive	64	64	1	none
***Staphylococcus pseudintermedius* Sps 3**								
oil (mg/mL)	4.8	2.4	0.5	0.625	11.2	1.4	0.125	0.188
gentamicin (µg/mL)	128	16	0.125	additive	64	4	0.0625	synergy
oil (mg/mL)	4.8	0.3	0.0625	0.563	11.2	11.2	1	2.0
enrofloxacin (µg/mL)	64	32	0.5	additive	64	64	1	none
***Staphylococcus pseudintermedius* Sps 4**								
oil (mg/mL)	4.8	1.2	0.25	0.5	5.6	1.4	0.250	0.375
gentamicin (µg/mL)	64	16	0.25	synergy	32	4	0.125	synergy
oil (mg/mL)	4.8	4.8	1	2.0	11.2	11.2	1	2.0
enrofloxacin (µg/mL)	32	32	1	none	64	64	1	none
***Staphylococcus pseudintermedius* Sps 5**								
oil (mg/mL)	4.8	1.2	0.25	0.5	44.8	11.2	0.25	0.281
gentamicin (µg/mL)	256	64	0.25	synergy	128	4	0.031	synergy
oil (mg/mL)	4.8	0.3	0.0625	0.563	11.2	5.6	0.5	1.0
enrofloxacin (µg/mL)	64	32	0.5	additive	64	32	0.5	additive
***Staphylococcus pseudintermedius* Sps 6**								
oil (mg/mL)	4.8	2.4	0.5	0.75	22.4	2.8	0.125	0.156
gentamicin (µg/mL)	128	32	0.25	additive	64	2	0.031	synergy
oil (mg/mL)	4.8	4.8	1	2.0	22.4	22.4	1	3.0
enrofloxacin (µg/mL)	32	32	1	none	64	128	2	none
***Staphylococcus pseudintermedius* Sps 7**								
oil (mg/mL)	4.8	2.4	0.5	1	5.6	1.4	0.250	0.375
gentamicin (µg/mL)	128	64	0.5	additive	32	4	0.125	synergy
oil (mg/mL)	4.8	0.08	0.016	0.516	11.2	5.6	0.5	1.0
enrofloxacin (µg/mL)	64	32	0.5	additive	128	64	0.5	additive
***Staphylococcus pseudintermedius* Sps 8**								
oil (mg/mL)	4.8	1.2	0.25	0.75	11.2	1.4	0.125	0.188
gentamicin (µg/mL)	128	64	0.5	additive	64	4	0.0625	synergy
oil (mg/mL)	4.8	4.8	1	2.0	11.2	11.2	1	2.0
enrofloxacin (µg/mL)	64	64	1	none	64	64	1	none
***Staphylococcus pseudintermedius* Sps 9**								
oil (mg/mL)	4.8	1.2	0.25		44.8	11.2	0.25	

antimicrobial agent	patchouli oil				tea tree oil			
	MICi	MICc	FIC	∑FIC [interaction]	MICi	MICc	FIC	∑FIC [interaction]
gentamicin (µg/mL)	64	8	0.125	0.375	32	4	0.125	0.375
oil (mg/mL)	**4.8**	**4.8**	1	2.0	**11.2**	**11.2**	1	2.0
enrofloxacin (µg/mL)	0.125	0.125	1	none	0.125	0.125	1	none
***Staphylococcus pseudintermedius* Sps 10**								
oil (mg/mL)	**4.8**	**1.2**	0.25	0.506	**11.2**	**2.8**	0.25**0**	0.5**06**
gentamicin (µg/mL)	0.125	0.032	0.256	additive	0.0625	0.016	0.256	additive
oil (mg/mL)	**4.8**	**4.8**	1	2.0	**11.2**	**11.2**	1	2.0
enrofloxacin (µg/mL)	64	64	1	none	32	32	1	none
***Staphylococcus pseudintermedius* Sps 11**								
oil (mg/mL)	**4.8**	**2.4**	0.5	0.75	**22.4**	**5.6**	0.25	0.378
gentamicin (µg/mL)	0.5	0.125	0.25	additive	0.125	0.016	0.128	synergy
oil (mg/mL)	**4.8**	**4.8**	1	2.0	**5.6**	**5.6**	1	2.0
enrofloxacin (µg/mL)	0.125	0.125	1	none	0.125	0.125	1	none
***Staphylococcus pseudintermedius* Sps 12**								
oil (mg/mL)	**4.8**	**2.4**	0.5	0.75	**44.8**	**11.2**	0.25	0.5
gentamicin (µg/mL)	32	8	0.25	additive	8	2	0.25	synergy
oil (mg/mL)	**4.8**	**4.8**	1	2.0	**11.2**	**11.2**	1	2.0
enrofloxacin (µg/mL)	0.5	0.5	1	none	0.5	0.5	1	none

Sa – *Staphylococcus aureus;* Sps – *Staphylococcus pseudintermedius*
MIC_i : MIC individually as $MIC^{(O)}$ or $MIC^{(A)}$; MIC_c : MIC in combination as $MIC^{(OxA)}$ or $MIC^{(AxO)}$; FIC: Fractional Inhibitory Concentrations
none = noninteractive
